# Intellectual Freedom and Crone Pedagogies

**DOI:** 10.1093/geroni/igaf122.438

**Published:** 2025-12-31

**Authors:** Nicholas DiCarlo

**Affiliations:** University of California, San Francisco, San Francisco, California, United States

## Abstract

Crone pedagogies (DiCarlo, 2023 & 2004) provide grounding frameworks for collective survival for teaching-learning. Re-membering the emancipatory potential of historical memory counters colonial hegemony’s ongoing assault on collective vitality and indigenous ways of knowing. Opposing the logics of extraction and exploitation, crone pedagogies invest in the collective by resisting disposability and neoliberalism’s insistence on competition and scarcity. The gerontological imagination (Estes, 1992) calls us to envision bridges between frameworks that understand how ideology exploits material reality (Estes, 1979) and shapes subjectivity (Gaztambide, 2023). These bridges equip scholars to sustain engagement with complex feelings about traumatic sociopolitical realities over time, pushing past analytic frames that comfort and quell and pursuing unsettling and disillusionment that antagonize institutional complicity (Layton, 2019).

